# Survey of infectious diseases providers reveals variability in duration of antibiotic therapy for the treatment of Gram-negative bloodstream infections

**DOI:** 10.1093/jacamr/dlac005

**Published:** 2022-02-09

**Authors:** Joshua T. Thaden, Pranita D. Tamma, Qing Pan, Yohei Doi, Nick Daneman

**Affiliations:** 1 Division of Infectious Diseases, Duke University School of Medicine, Durham, NC, USA; 2 Department of Pediatrics, Johns Hopkins University School of Medicine, Baltimore, MD, USA; 3 Department of Statistics, The George Washington University, Washington, DC, USA; 4 Center for Innovative Antimicrobial Therapy, Division of Infectious Diseases, University of Pittsburgh School of Medicine, Pittsburgh, PA, USA; 5 Departments of Microbiology and Infectious Diseases, Fujita Health University School of Medicine, Toyoake, Aichi, Japan; 6 Sunnybrook Health Sciences Centre, University of Toronto, Toronto, Ontario, Canada

## Abstract

**Background:**

Trials supporting shorter durations of antibiotic therapy for Gram-negative bloodstream infections (GN-BSI) have recently been published. However, adoption of these findings into practice is unclear given limited eligibility criteria and relatively large non-inferiority margins of these studies. To better understand contemporary management of GN-BSI, we conducted an international survey of infectious diseases (ID) specialists.

**Methods:**

We developed and disseminated an online survey to assess practice patterns involving treatment duration of GN-BSI, including providers from 28 countries. χ^2^ tests, *t*-tests and multivariable linear regression with generalized estimating equations were used to identify factors associated with treatment duration.

**Results:**

In total, 277 ID specialists completed the survey (64% physicians, 31% pharmacists). The median reported duration of antibiotics was 7 days (IQR, 7–10 days) for all GN-BSI sources. Thirty percent of providers typically recommend durations that differ by ≥7 days depending on the source of GN-BSI, and 71% treat ≥10 days for at least one source. In an adjusted model, factors associated with increased duration included intra-abdominal (+1.01 days, 95% CI 0.57–1.45 days; *P *< 0.0001), vascular catheter (+0.74 days; 0.33–1.15 days; *P *= 0.0004), and respiratory (+0.76 days; 0.38–1.14 days; *P *< 0.0001) sources of GN-BSI relative to urinary sources. Providers that transition patients to oral therapy report shorter durations than those who treat with full IV therapy (−0.60 days; −1.12 to −0.09 days; *P *= 0.02).

**Conclusions:**

There is extensive heterogeneity in duration of therapy for treating GN-BSI, particularly with respect to source of GN-BSI. Investigations into appropriate treatment durations for different GN-BSI sources are needed.

## Introduction

Gram-negative bloodstream infections (GN-BSI) are common and associated with high morbidity and mortality.^1,2^ Three published randomized controlled trials (RCTs) support shorter durations of therapy than historically prescribed for GN-BSI,^3–5^ though they had relatively large non-inferiority margins and limited external validity due to lack of immunocompromised patients and non-fermenting Gram-negative bacterial infections (e.g. *Pseudomonas aeruginosa*). Large non-inferiority margins may make it more difficult to detect real differences in clinical outcomes between treatment groups, and limited eligibility criteria can hinder our ability to generalize results to broad patient populations. It is unknown if the results of these trials have been adopted into clinical practice as there may be concerns about their applicability to the subpopulations of patients excluded or underrepresented in clinical trials, there may not be awareness of the clinical trials, or because it is difficult to change longstanding views that prolonged durations of therapy are necessary to treat GN-BSIs. There are many potential benefits of treating GN-BSI with shorter antibiotic durations, including decreased adverse drug events and *Clostridioides difficile* infections, decreased length of hospital stay and decreased complications from a vascular catheter such as catheter occlusion, venous thrombosis, phlebitis, extravasation and catheter-associated bloodstream infections.^3,6–8^ Through the development and dissemination of an international survey of infectious diseases (ID) specialists, we sought to describe contemporary practices surrounding the duration of therapy for managing GN-BSI and to understand the residual heterogeneity in treatment recommendations.

## Methods

### Survey development and content

A 41 question multiple choice and open-ended question online survey was developed by the authors, with pilot and sensibility testing by ID physicians and pharmacists with expertise in managing GN-BSI. Participants were requested to enter demographic data (e.g. professional title, country of practice, years of experience). The majority of the survey questions focused on management decisions for each of five scenarios of GN-BSI: pneumonia, vascular catheter infection, urinary tract infection (UTI), intra-abdominal infection (IAI) and skin/soft tissue infection (SSTI). Survey questions focused on patients without severe immunocompromise. For each source of GN-BSI, respondents answered the following general questions: (i) typical recommended treatment duration; (ii) factors influencing treatment duration (e.g. antibiotic resistance, bacterial species, source control [vascular catheter infection and IAI only]); (iii) whether they typically transition patients to oral antibiotic therapy; (iv) minimum duration of IV therapy prior to oral step-down therapy; and (v) factors influencing the decision to step down to oral antibiotics (e.g. afebrile, normotensive, concern for poor oral absorption, normal white blood cell, knowledge of the percent bioavailability and likelihood of sustained serum concentrations of oral agents, negative follow-up blood cultures, bacterial species, source control). Survey results focused on oral step-down were previously published.^9^ The complete survey is available in Appendix S1 (available as Supplementary data at *JAC-AMR* Online).

### Survey distribution

The survey was distributed by a link emailed to organizational listservs or message boards of international ID physicians and pharmacists. These organizations included the American College of Clinical Pharmacy, Antibacterial Resistance Leadership Group, Asia Pacific Foundation for Infectious Diseases, Australasian Society for Infectious Diseases, European Society of Clinical Microbiology and Infectious Diseases, Federation of Infectious Diseases in South Africa, Infectious Diseases Society of America, South African Society of Clinical Pharmacy, South African Society of Hospital Pharmacists, and Society of Hospital Pharmacists of Australia. The survey was opened on 28 October 2020 and closed on 17 December 2020. The survey link was emailed to potential respondents one time.

### Definitions

Treatment duration range was calculated for each survey respondent by subtracting their minimum treatment duration recommendation from their maximum treatment duration recommendation across the five GN-BSI sources. The long duration subgroup was defined as providers that typically treat all five GN-BSI sources (pneumonia, vascular catheter, urine, IAI, SSTI) with ≥10 days of antibiotics.

### Statistics

Only respondents that completed the entire survey were included in the final analysis. Descriptive statistics included percentage for each response category, mean (SD), or median (IQR). Proportions were compared with χ^2^ tests. Means of continuous variables were compared with *t*-tests. Variables associated with durations of antibiotic therapy were investigated using generalized estimating equations (GEEs) with identity link function to account for clustering within respondents (given that each respondent provided recommendations for five different scenarios).^10,11^ An exchangeable correlation structure was specified. Variables adjusted for in the GEE model were pre-specified based on clinical relevance, and included provider demographics (position, experience, location of practice [i.e. US versus ex-US]), bloodstream infection source and whether a provider typically steps down to oral therapy for the bloodstream infection source. Furthermore, candidate risk factors for routinely prescribing prolonged durations of antibiotic therapy were investigated using logistic regressions. Statistical analyses were performed using GraphPad Prism version 9 (San Diego, CA, USA) and RStudio version 1.3.1073 (Boston, MA, USA).

## Results

### Demographics of survey respondents

In total, 329 respondents started the survey and 277 completed the survey (277/329 [84%]). There were no significant demographic differences between those that did and did not complete the survey (data not shown). The remainder of the manuscript will focus on those that completed the survey. The majority of respondents were ID physicians (176/277 [64%]) or ID pharmacists (86/277 [31%]) (Figure S1). The location of respondents was as follows: North America (201/277 [73%]), Asia (36/277 [13%]) and Africa (19/277 [7%]). There was variability in the respondents’ years of experience since terminal degree (e.g. MD, PharmD) as 104 (38%) respondents practiced as independent ID specialists for 1–10 years, 97 (35%) practiced for 11–20 years, 39 (14%) practiced for 21–30 years, and 37 (13%) practiced for more than 30 years.

### Duration of therapy

**Figure 1.**
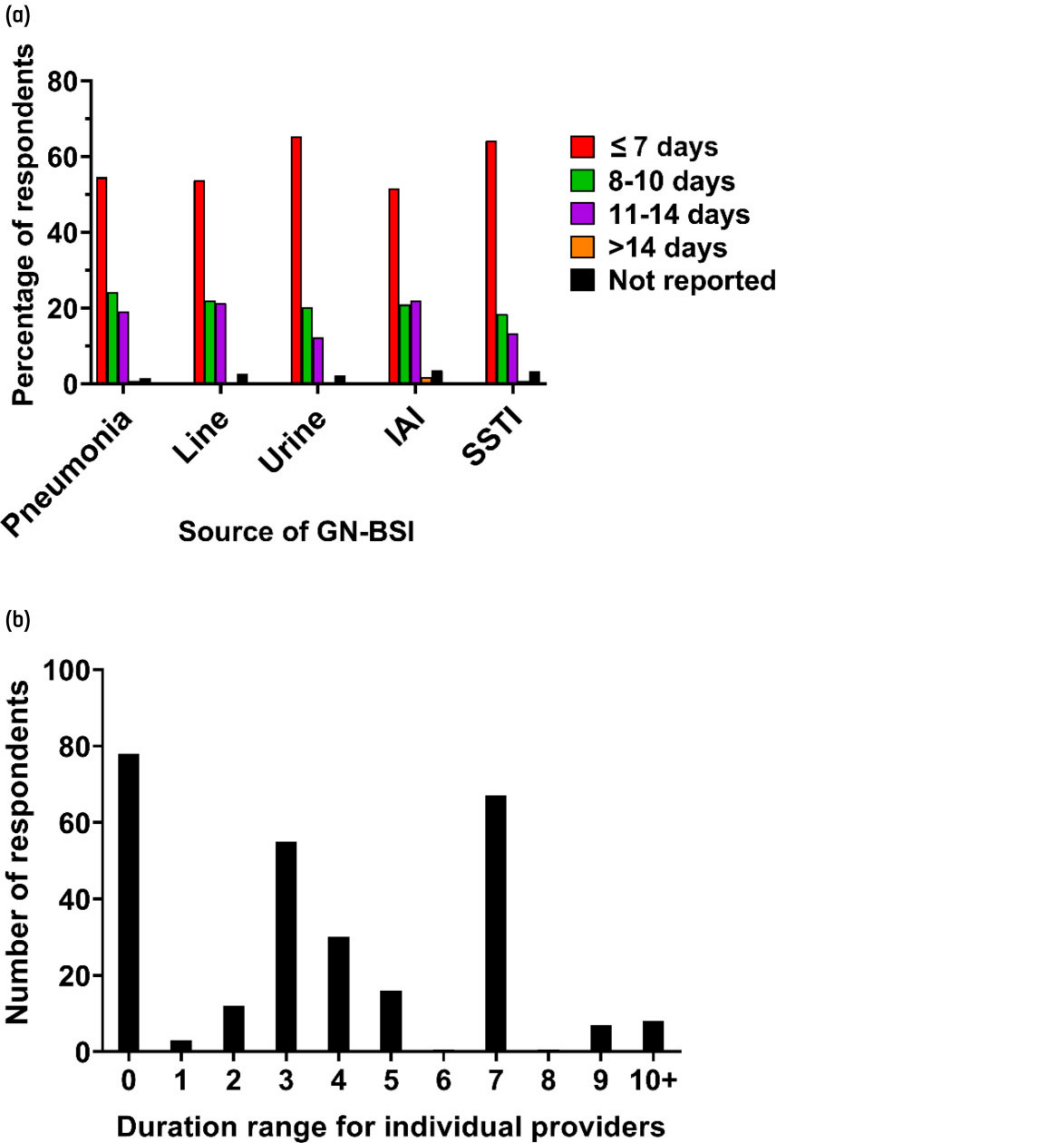
(a) Typical recommended duration of antibiotic therapy in GN-BSI. For each source of GN-BSI, the percentage of providers that treat for each duration group is shown. (b) Distribution of provider treatment range. For each provider, a treatment duration range was calculated as the difference between their maximum and minimum recommended duration across the five syndromes. Line, vascular catheter infection.

For all sources of GN-BSI, the median preferred duration of therapy was 7 days (IQR, 7–10 days), though the distributions of durations between sources were not equivalent (*P *= 0.0001) (Figure 1a). The percentage of providers that reported treating GN-BSI for ≤7 days ranged from 52% (143/277) for bacteraemic IAIs to 65% (181) for bacteraemic UTIs. For individual providers, significant variation was observed in the treatment duration between GN-BSI sources. Twenty-eight percent of providers reported prescribing the same duration of therapy regardless of the source of infection, while 30% of providers reported duration ranges of ≥7 days depending on the source of infection (Figure 1b). The majority (198/277 [72%]) of providers reported a duration of ≥10 days for at least one GN-BSI source.

**Figure 2.**
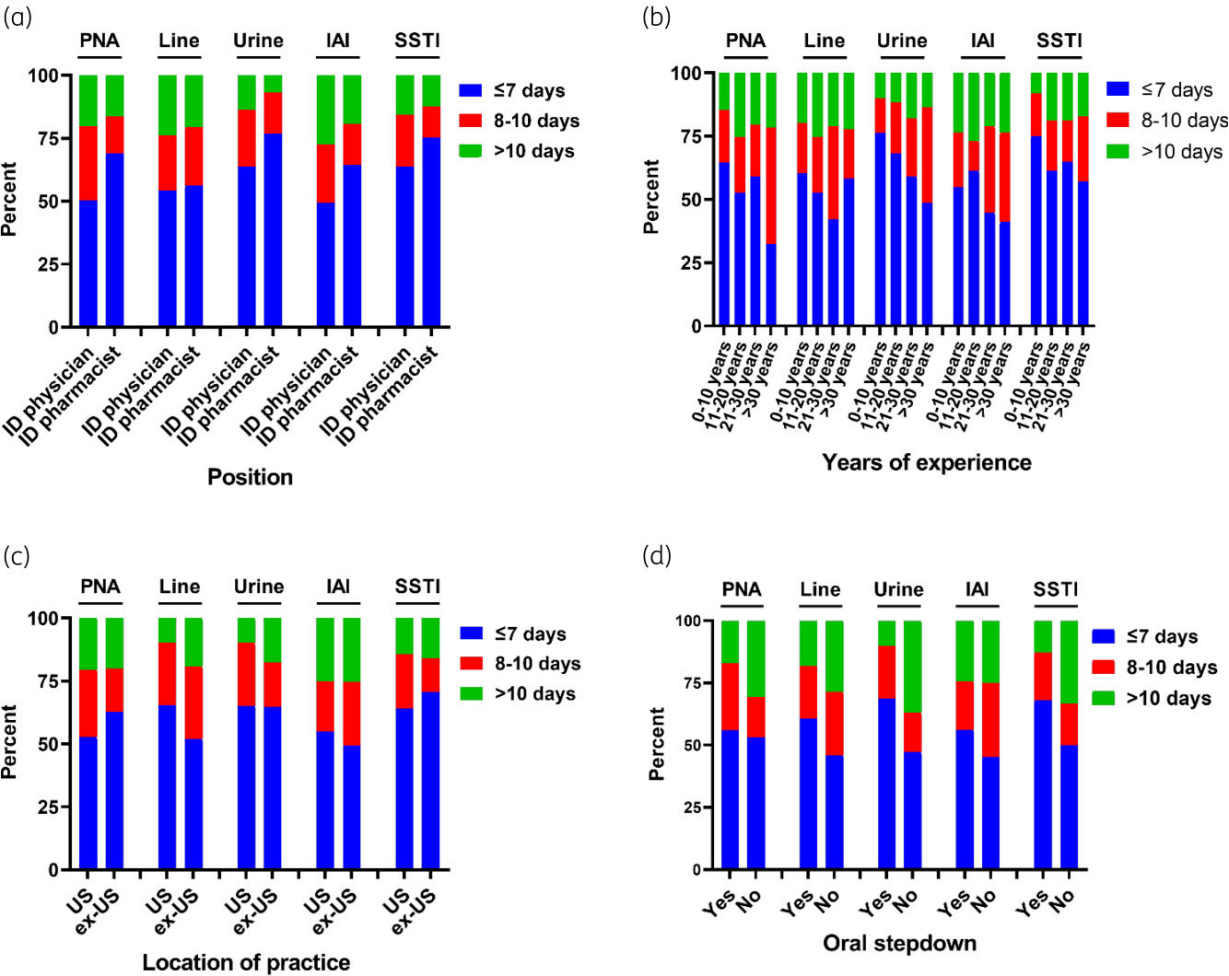
Typical duration of antibiotic therapy in managing GN-BSI from survey respondents. The data were stratified by position (ID physician versus ID pharmacist) (a), years of experience since terminal degree (e.g. MD, PharmD) (b), location of practice (within USA [US] versus outside USA [ex-US]) (c), and whether the provider typically steps down to oral therapy in managing GN-BSI from the particular source of GN-BSI (d). Line, vascular catheter infection; PNA, pneumonia.

Preferred duration of therapy stratified by position, years of experience, location of practice and whether the provider typically practices step-down to oral therapy for the GN-BSI source are shown in Figure 2(a)–(d), respectively. Stratification by provider position revealed that ID pharmacists were likely to recommend shorter durations of therapy for multiple GN-BSI sources compared with ID physicians (pneumonia: mean 8.8 days for ID pharmacists versus 9.3 days for ID physicians, *P *= 0.23; vascular catheter: 9.2 days versus 9.2 days, *P *= 0.99; UTI: 7.6 days versus 8.6 days, *P *= 0.0005; IAI: 8.7 days versus 9.6 days, *P *= 0.03; SSTI: 8.2 versus 8.6 days, *P *= 0.21). Stratification by whether providers typically transition to oral therapy revealed that oral step-down was associated with shorter duration of therapy for some GN-BSI sources (respiratory: mean 9.0 days for oral step-down group versus 9.5 days for full IV group, *P *= 0.20; vascular catheter: 8.8 days versus 9.7 days, *P *= 0.03; UTI: 8.1 days versus 8.7 days, *P *= 0.11; IAI: 9.3 days versus 9.4 days, *P *= 0.77; SSTI: 8.3 days versus 10.4 days, *P *= 0.05).

We further examined the variables associated with duration of therapy using GEE models. In univariable GEE models, covariates including IAI (+1.11 days; 95% CI 0.68 to 1.53 days; *P *< 0.00001), vascular catheter (+0.92 days; 95% CI 0.52 to 1.31 days; *P *< 0.00001) and respiratory (+0.85 days; 95% CI 0.48 to 1.21 days; *P *< 0.00001) sources of GN-BSI were associated with increased duration of therapy for GN-BSI, while ID pharmacist position (−0.56 days; 95% CI −1.11 to −0.01 days; *P *= 0.05) and oral step-down (−0.91 days; 95% CI −1.37 to −0.44 days; *P *= 0.0001) were associated with decreased durations of therapy (Table 1). In the multivariable GEE model, IAI (+1.01 days, 95% CI 0.57–1.45 days; *P *< 0.0001), vascular catheter (+0.74 days; 95% CI 0.33–1.15 days; *P *= 0.0004), and respiratory (+0.76 days; 95% CI 0.38–1.14 days; *P *< 0.0001) sources of GN-BSI were associated with longer durations of therapy relative to urinary sources, while providers likely to step down to oral antibiotic therapy were likely to prescribe a decreased duration of therapy (−0.60 days; 95% CI −1.12 to −0.09 days; *P *= 0.02) (Table 1).

**Table 1.** Prescriber and patient characteristics associated with duration of therapy

Variable	Univariable analysis			Multivariable analysis		
	Coefficient estimate (days)	95% CI (days)	*P* value	Coefficient estimate (days)	95% CI (days)	*P* value
Position^a^						
ID pharmacist	−0.56	−1.11 to −0.01	**0**.**05**	−0.42	−1.00 to 0.16	0.15
Other	−0.43	−1.76 to 0.91	0.53	−0.50	−1.81 to 0.82	0.45
years of experience^b^						
11–20 years	0.52	−0.08 to 1.12	0.09	0.42	−0.16 to 1.01	0.15
21–30 years	0.60	−0.20 to 1.40	0.14	0.45	−0.36 to 1.26	0.27
>30 years	0.62	−0.18 to 1.42	0.13	0.45	−0.36 to 1.26	0.27
Practice in USA^c^	0.10	−0.49 to 0.70	0.12	0.04	−0.62 to 0.70	0.90
Source of GN-BSI^d^						
IAI	1.11	0.68 to 1.53	**<0**.**00001**	1.01	0.57 to 1.45	**<0**.**00001**
vascular catheter	0.92	0.52 to 1.31	**<0**.**00001**	0.74	0.33 to 1.15	**0**.**0004**
pneumonia	0.85	0.48 to 1.21	**<0**.**00001**	0.76	0.38 to 1.14	**<0**.**00001**
SSTI	0.27	−0.07 to 0.62	0.12	0.27	−0.08 to 0.61	0.13
Oral step-down^e^	−0.91	−1.37 to −0.44	**0**.**0001**	−0.60	−1.12 to −0.09	**0**.**02**

Statistically significant values are highlighted in bold.

a Reference group is ID physician.

b Reference group is 0–10 years of experience.

c Reference group is providers that practice outside the USA.

d Reference group is urinary tract source of infection.

e Reference group is providers that do not step down to oral therapy.

### Long duration subgroup

In order to understand factors associated with providers that typically recommend longer courses of therapy across the full range of GN-BSI sources, we identified a long duration subgroup of providers that typically treat ≥10 days for all GN-BSI sources. Fifteen percent (42/277) of providers were in the long duration subgroup. The mean duration of therapy in the long duration group was 13.1 days (SD 3.0 days) for pneumonia, 12.3 days (SD 2.0) for vascular catheter, 11.6 days (SD 2.0) for urine, 13.1 days (SD 2.6) for IAI and 11.9 days (SD 2.0) for SSTI sources of GN-BSI. Ultimately no provider demographic variables were significantly associated with membership in the long duration subgroup when analysed with χ^2^ tests (position: *P *= 0.50; experience: *P *= 0.54; geography: *P *= 0.52; whether transition to oral antibiotics for all GN-BSI sources or not: *P *= 0.83) (Figure S2). Univariable and multivariable logistic regression analyses similarly did not reveal an association between provider demographics or oral step-down practices and the long duration subgroup (data not shown).

### Surveyed factors that influence treatment duration

**Figure 3.**
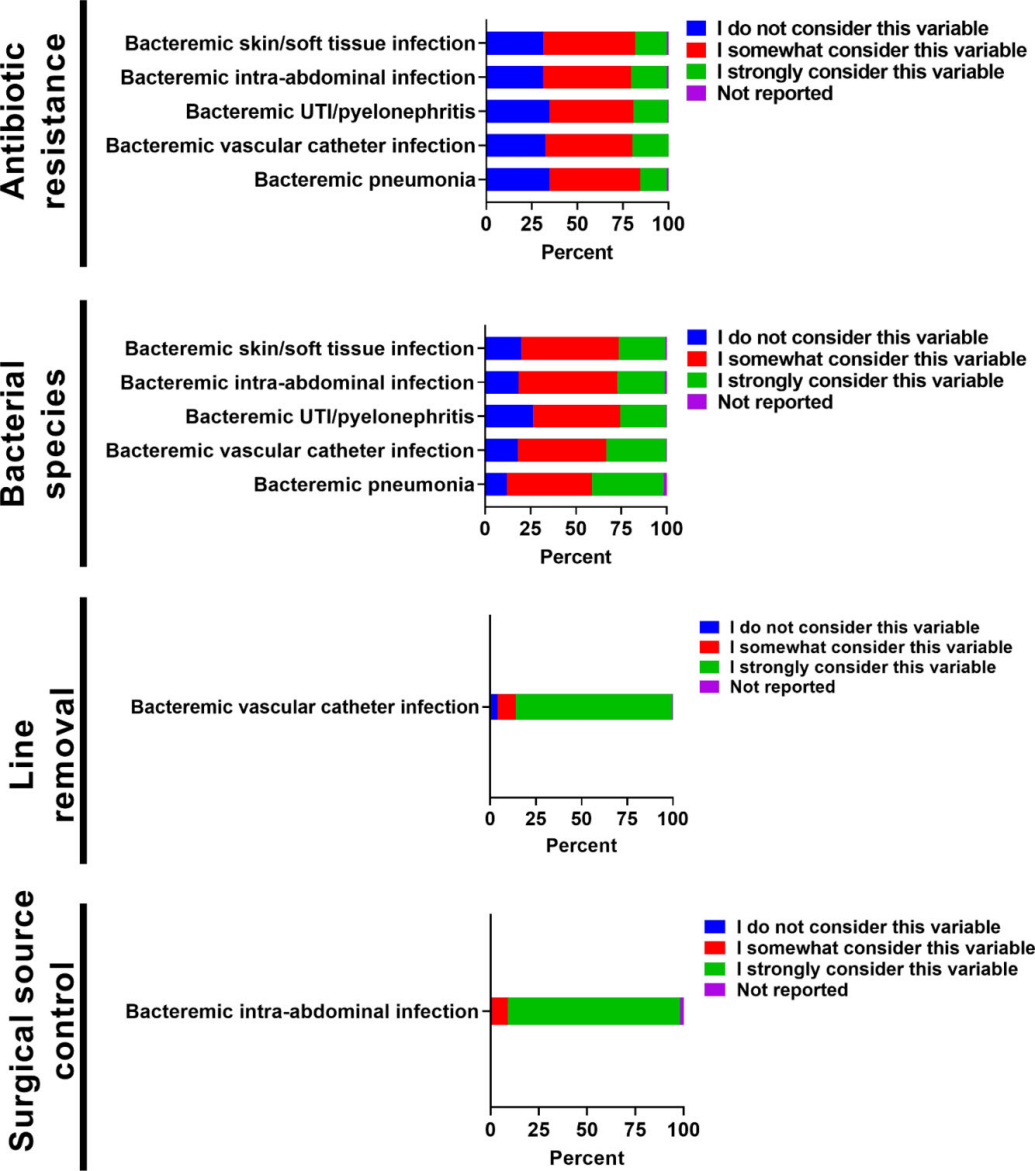
Factors that influence duration of antibiotic therapy. Providers were surveyed on how strongly they consider each listed variable before determining duration of therapy in treating GN-BSI.

Survey participants were asked to consider how strongly they consider certain factors in the duration of therapy decision (Figure 3). Antibiotic resistance was strongly considered in the treatment duration decision by 15%–20% of respondents to each of the GN-BSI scenarios. Bacterial species (e.g. *Escherichia coli* versus *P. aeruginosa*) was strongly considered by 25%–39% of providers. For vascular catheter infections, line removal was strongly considered by 86% of providers. Similarly, for IAIs, surgical source control was a factor strongly considered by 89% of providers. Factors that influence treatment duration were generally similar between ID physicians and ID pharmacists, with few exceptions (Figure S3).

## Discussion

One of the key management decisions for GN-BSI is the duration of antibiotic therapy. We conducted a survey of provider practice patterns involving this treatment decision to identify areas of controversy that remain, despite the existence of three clinical trials (only two of which were published at the time of survey distribution^3,4^). There were several important findings from this work. First, despite clinical trials suggesting that shorter durations of therapy (i.e. 7 days) are non-inferior to longer therapy (i.e. 14 days),^3–5^ only approximately half of survey respondents report adopting this practice. The incomplete adoption of recent trial results is not surprising given that prior work has shown that it may take >10 years for research findings to be incorporated into clinical practice.^12,13^ In addition, the three clinical trials investigating this question had stringent eligibility criteria, as is generally the case for randomized controlled trials, potentially limiting their generalizability; >75% of patients with GN-BSI that were screened for the three published trials were not ultimately enrolled. It can be challenging to apply the results of clinical trials to a broad array of patients. For example, existing trials have focused on non-critically ill patients that have demonstrated clinical stability prior to randomization.^3–5^ However, observational studies with less restrictive inclusion/exclusion have also supported shorter duration of therapy.^14–16^ The currently published trials also included large absolute non-inferiority margins (10%)^3–5^ that in some cases are higher than the absolute event rate in the comparator group,^4,5^ or focused on duration of antibiotic therapy as the primary endpoint.^5^ Some prescribers may be unwilling to change practice until larger trials have established non-inferiority of shorter durations with greater precision (i.e. smaller non-inferiority margins).^17^ Nevertheless, the duration of therapy for GN-BSI appears to be decreasing over time. For example, 52%–65% of providers (depending on the source of GN-BSI) typically reported treating for ≤7 days in our survey conducted in 2020, compared with 11% in a 2018 survey.^18^ And the typical duration of therapy in this study is also lower than noted in recent European surveys where *E. coli* and *P. aeruginosa* bloodstream infection were treated for median durations of 10 and 10–14 days, respectively.^19,20^ In addition, historical observational cohorts of patients with GN-BSI indicate higher median durations of therapy (≥10 days) than that reported in this survey.^15,21,22^

Second, we identified considerable intra- and inter-provider variability in duration of antibiotic therapy for treating GN-BSI. Individual providers varied greatly in their typical treatment duration. Approximately a third of individual providers had treatment durations that varied by ≥7 days depending on the source of the GN-BSI, and 71% of providers typically treat at least one source of GN-BSI for ≥10 days. IAI, vascular catheter and respiratory sources of GN-BSI were associated with longer durations of therapy. There may be concern for inadequate source control in these infections as this variable was strongly considered in the duration decision by the surveyed providers. Interestingly, factors such as bacterial species and antibiotic resistance were not strongly considered in the duration decision by most surveyed providers. This is despite the fact that patients with GN-BSI due to non-Enterobacterales (e.g. *P. aeruginosa*, *Acinetobacter baumannii*, *Stenotrophomonas maltophilia*) or highly antibiotic-resistant organisms (e.g. carbapenem resistant) were not well-represented in the clinical trials that address duration of therapy.^3–5^ In terms of inter-provider variability, we found that respondents that treat with longer durations of therapy are also more likely to pursue IV therapy for a complete treatment course. However, we did not identify any provider demographics associated with the long duration subgroup (i.e. typically treat for ≥10 days regardless of GN-BSI source).

This study has several limitations. First, we are unable to quantify the non-response bias in this voluntary online survey. Providers that either did not have strong feelings about these management decisions or were too busy to respond could have influenced the results. Second, providers who do not belong to professional organizations would not have been contacted to complete this survey. It is unclear how practices surrounding the management of GN-BSI might differ among providers who do and do not belong to professional organizations. Third, there may have been response bias. The survey relies on self-report, and we cannot verify that the providers’ responses reflect their actual clinical practice. Finally, only 27% of respondents practiced outside the USA, and this limited our ability to fully explore the factors driving differences in US versus ex-US providers, including regions of the world that were underrepresented such as Latin America. The survey was only provided in English, and this may have limited the response where English is not the primary language.

Prior surveys of GN-BSI clinical practice patterns have been published;^18–20^ however, we believe that this work makes additional contributions to the literature for several reasons. Our survey involved participants from six continents and multiple ID professions (e.g. ID physicians, ID pharmacists) who varied widely in years of experience. Additionally, our survey captured a granular level of detail that allowed for a comprehensive understanding of intra- and inter-provider variability in practice patterns. Although evidence supporting shorter duration of treatment for GN-BSI has begun to emerge, there remains wide variation in reported antibiotic treatment duration across ID specialists. There is also wide variation within individual providers’ practice, with most still recommending prolonged treatment for at least one of the common GN-BSI syndromes. Additional RCTs increasingly representative of clinical practice will clarify the optimal duration of therapy for managing GN-BSI.

## Supplementary Material

dlac005_Supplementary_Data

## Supplementary data

Appendix S1 and Figures S1 to S3 are available as Supplementary data at *JAC-AMR* Online.
