# Supplementary material for: Survey of infectious diseases providers reveals variability in duration of antibiotic therapy for the treatment of Gram-negative bloodstream infections
Source: JAC Antimicrob Resist. 2022 Feb 9;4(1):dlac005. doi: 10.1093/jacamr/dlac005 (PMC8827556; doi:10.1093/jacamr/dlac005)
Supplement: dlac005_Supplementary_Data [file dlac005_supplementary_data.docx]

**Supplementary data**

**Figure S1. Demographics of survey respondents.** Data on position, location of practice, and years of experience were captured from each survey respondent. For position and location of practice, the total number of respondents in each category are listed in the legend. Years of experience refers to years since terminal degree (e.g., M.D., PharmD). Abbreviations: APP, advanced practice provider; ID, infectious diseases.

**
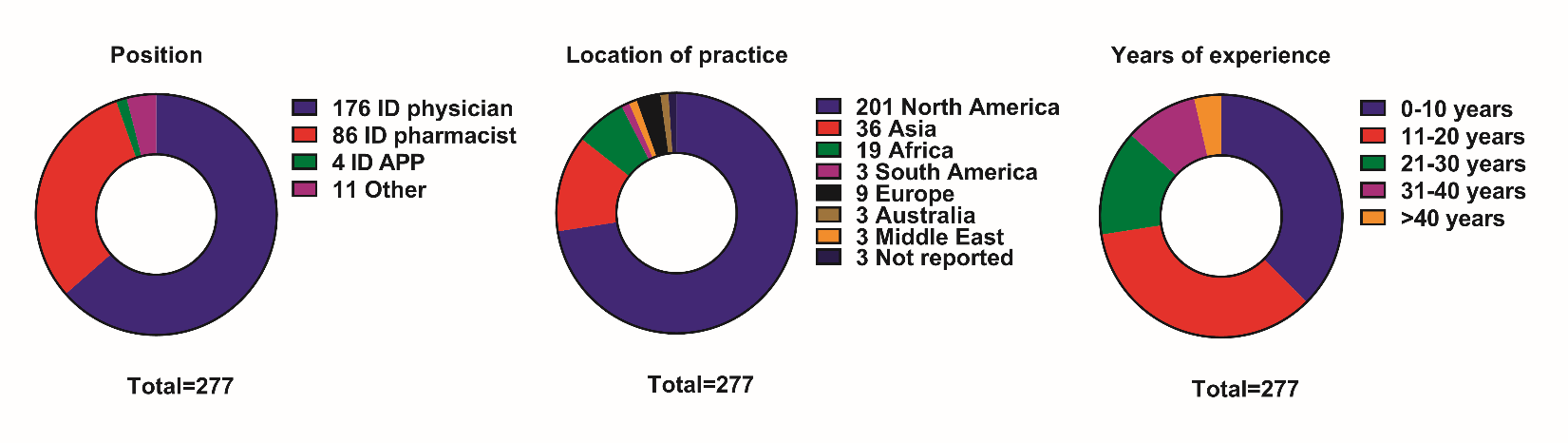
**

**Figure S2.** Demographics of providers in the long duration subgroup (i.e., typically treat GN-BSI from all five sources for ≥10 days) relative to all other providers, stratified by position (A), years of experience (B), geography (i.e., practice in the United State [US] or outside the US [ex-US]) (C), and whether the provider typically steps down to oral antibiotic therapy for all GN-BSI sources or not (D). P-values determined by chi-square tests.

**
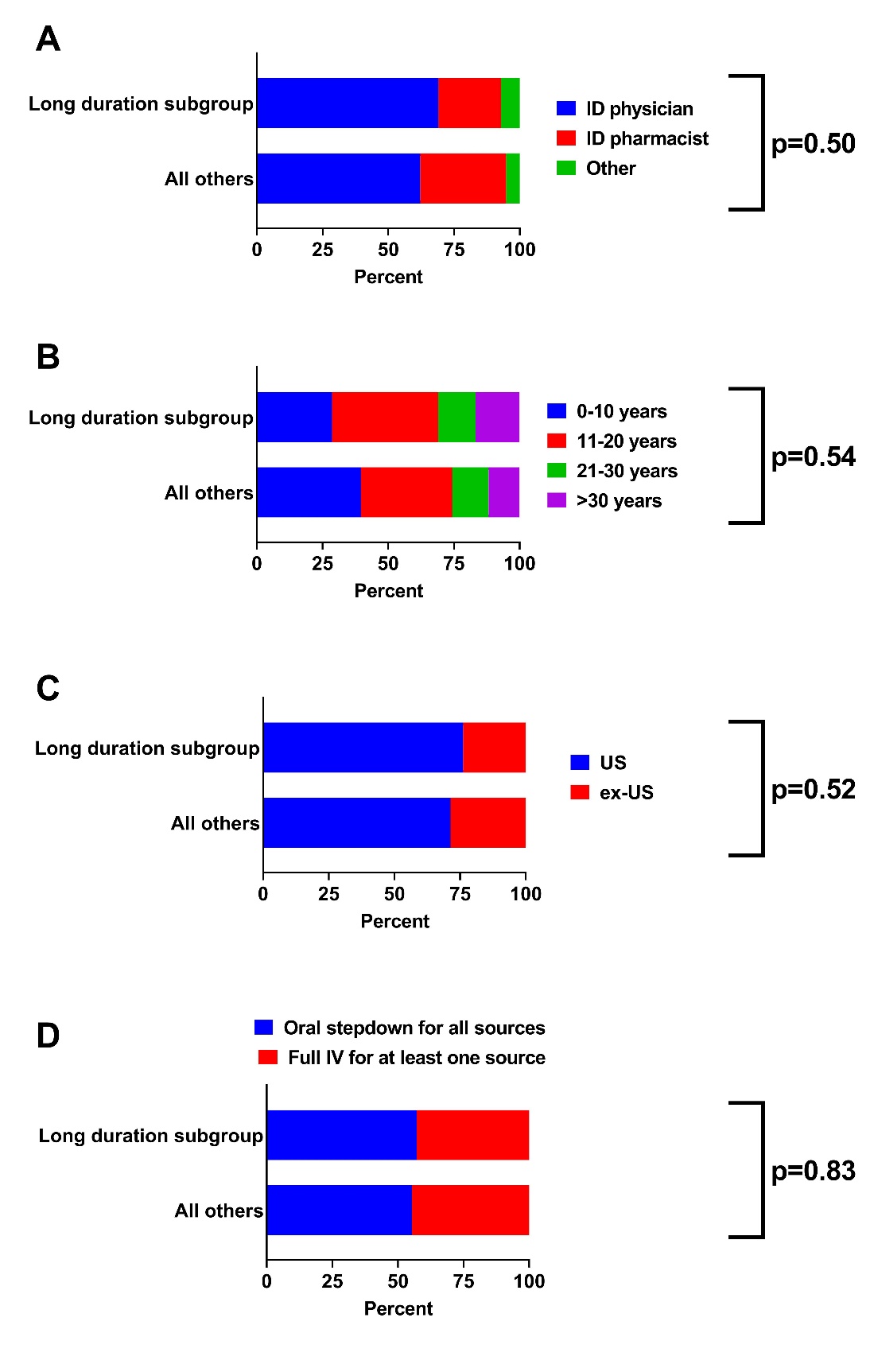
**

**Figure S3. Factors that influence the decision on duration of antibiotic therapy for managing Gram-negative bloodstream infection.** Providers were surveyed on how strongly they consider each listed variable before determining duration of antibiotics. The data was stratified by type of provider (i.e., ID physician versus ID pharmacist). Statistically significant differences (p<0.05) in the distribution of responses between physicians and pharmacists are noted by an asterisk (*). Abbreviations: IAI, intra-abdominal infection; ID, infectious diseases; Line, vascular catheter infection; SSTI, skin/soft tissue infection.

**
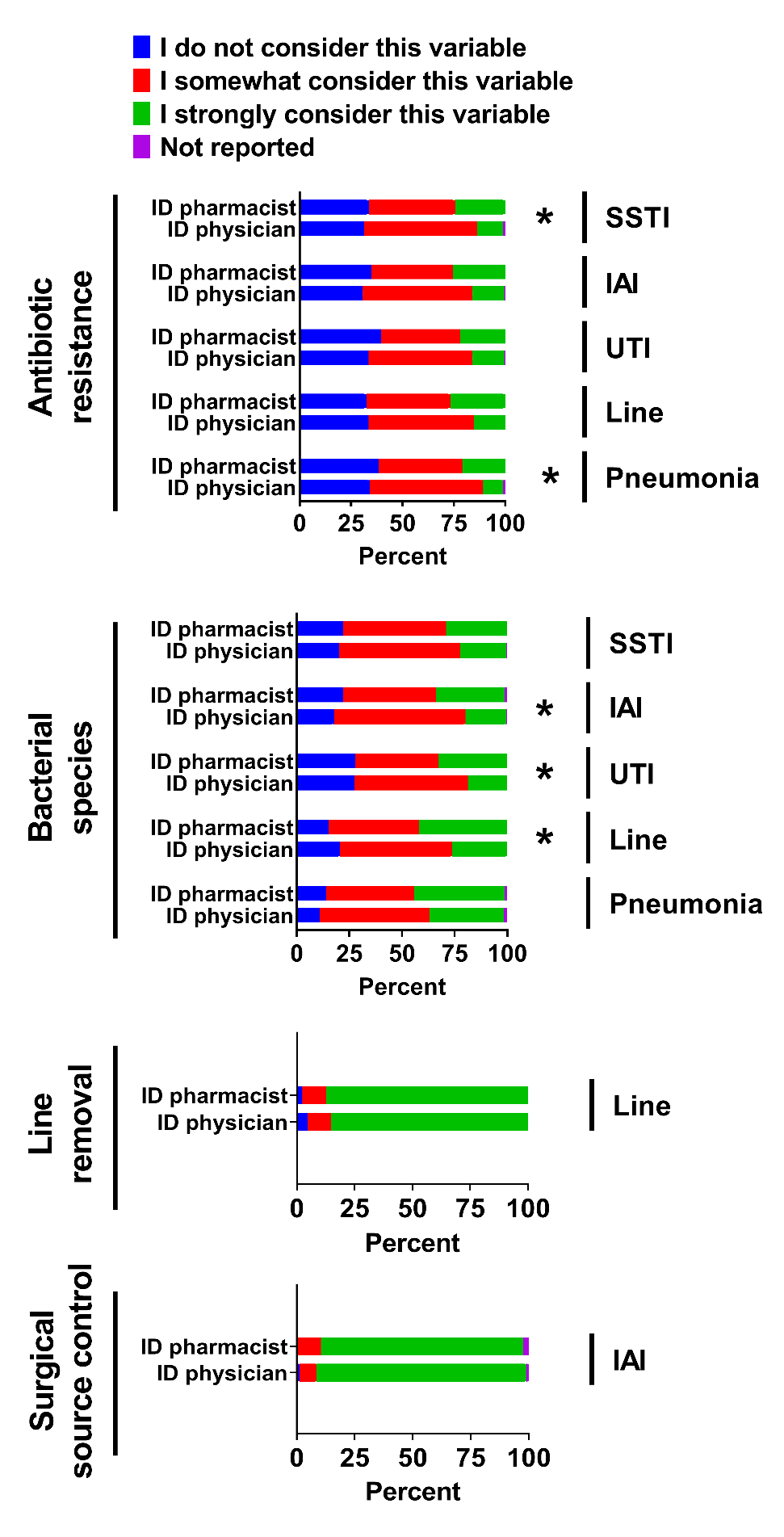
**

**Appendix S1. Schematic of online survey.** Providers were surveyed on both their typical oral stepdown practices and duration of antibiotic therapy practices in managing Gram-negative bacteremia. Data on oral stepdown practices was previously published (Thaden, Tamma, Doi, & Daneman, Int J Antimicrob Agents. 2021 Dec; 58(6):106451).

**Practice patterns in managing gram-negative bacteremia**

- Position: (Dropdown box with ID physician / ID advanced practice provider / ID pharmacist / Other)
  - If ‘Other’: What is your position? (text box)
- In what country do you primarily practice? (Dropdown box with countries listed)
  - If ‘Other’: In what country do you practice? (text box)
- How many years since you completed your degree (e.g., MD, PharmD, NP, PA)? (Dropdown list with 0-10, 11-20, 21-30, 31-40, >40)

**Bacteremic pneumonia**

- For gram-negative bacteremic pneumonia in patients that are not severely immunocompromised, what is your typical treatment duration? (Dropbox with numbers 0, 1, 2, 3, 4, …, 28+ days)
  - Please indicate whether the individual criteria below influence your treatment duration
    - Antibiotic resistance pattern of pathogen (I do not consider this variable at all in my duration decision / This variable is somewhat considered in my duration decision / This variable is strongly considered in my duration decision)
    - Species of gram-negative bacteria (e.g., *Escherichia coli* versus *Pseudomonas aeruginosa*) (I do not consider this variable at all in my duration decision / This variable is somewhat considered in my duration decision / This variable is strongly considered in my duration decision)
- Do you typically treat with IV antibiotics for the entire course? (yes/no)
  - If no: Is there a minimum duration of IV antibiotics you provide (yes/no)
    - If yes: What is the duration? (number)
  - If no: Please indicate whether individual criteria below influence decision to stepdown to oral therapy
    - Afebrile (I do not consider this variable at all in my stepdown decision / This variable is considered but not an absolute requirement prior to stepdown / This variable is an absolute requirement prior to stepdown)
    - Normotensive (I do not consider this variable at all in my stepdown decision / This variable is considered but not an absolute requirement prior to stepdown / This variable is an absolute requirement prior to stepdown)
    - Concern for poor oral absorption (I do not consider this variable at all in my stepdown decision / This variable is considered but not an absolute requirement prior to stepdown / This variable is an absolute requirement prior to stepdown)
    - Normal WBC (I do not consider this variable at all in my stepdown decision / This variable is considered but not an absolute requirement prior to stepdown / This variable is an absolute requirement prior to stepdown)
    - Bioavailability of the oral agent (I do not consider this variable at all in my stepdown decision / This variable is considered but not an absolute requirement prior to stepdown / This variable is an absolute requirement prior to stepdown)
    - Negative follow-up blood cultures (I do not consider this variable at all in my stepdown decision / This variable is considered but not an absolute requirement prior to stepdown / This variable is an absolute requirement prior to stepdown)
    - Whether gram-negative bacteria is an Enterobacterales (e.g., *Escherichia coli, Klebsiella* species*,* *Enterobacter* species), as opposed to other gram-negative bacteria such as *Pseudomonas aeruginosa* (I do not consider this variable at all in my stepdown decision / This variable is considered but not an absolute requirement prior to stepdown / This variable is an absolute requirement prior to stepdown)

**Bacteremic central line infection**

- For gram-negative bacteremic central line infections in patients that are not severely immunocompromised, what is your typical treatment duration? (Dropbox with numbers 0, 1, 2, 3, 4, …, 28+ days)
  - Please indicate whether the individual criteria below influence treatment duration
    - Antibiotic resistance pattern of pathogen (I do not consider this variable at all in my duration decision / This variable is somewhat considered in my duration decision / This variable is strongly considered in my duration decision)
    - Species of gram-negative bacteria (e.g., *Escherichia coli* versus *Pseudomonas aeruginosa*) (I do not consider this variable at all in my duration decision / This variable is somewhat considered in my duration decision / This variable is strongly considered in my duration decision)
    - Whether or not line is retained (I do not consider this variable at all in my duration decision / This variable is somewhat considered in my duration decision / This variable is strongly considered in my duration decision)
- Do you typically treat with IV antibiotics for the entire course? (yes/no)
  - If no: Is there a minimum duration of IV antibiotics you provide (yes/no)
    - If yes: What is the duration? (number)
  - If no: Please indicate whether individual criteria below influence decision to stepdown to oral therapy
    - Afebrile (I do not consider this variable at all in my stepdown decision / This variable is considered but not an absolute requirement prior to stepdown / This variable is an absolute requirement prior to stepdown)
    - Normotensive (I do not consider this variable at all in my stepdown decision / This variable is considered but not an absolute requirement prior to stepdown / This variable is an absolute requirement prior to stepdown)
    - Concern for poor oral absorption (I do not consider this variable at all in my stepdown decision / This variable is considered but not an absolute requirement prior to stepdown / This variable is an absolute requirement prior to stepdown)
    - Normal WBC (I do not consider this variable at all in my stepdown decision / This variable is considered but not an absolute requirement prior to stepdown / This variable is an absolute requirement prior to stepdown)
    - Bioavailability of the oral agent (I do not consider this variable at all in my stepdown decision / This variable is considered but not an absolute requirement prior to stepdown / This variable is an absolute requirement prior to stepdown)
    - Negative follow-up blood cultures (I do not consider this variable at all in my stepdown decision / This variable is considered but not an absolute requirement prior to stepdown / This variable is an absolute requirement prior to stepdown)
    - Whether gram-negative bacteria is an Enterobacterales (e.g., *Escherichia coli, Klebsiella* species*,* *Enterobacter* species), as opposed to other gram-negative bacteria such as *Pseudomonas aeruginosa* (I do not consider this variable at all in my stepdown decision / This variable is considered but not an absolute requirement prior to stepdown / This variable is an absolute requirement prior to stepdown)
    - The line is removed within the first few days of diagnosis (I do not consider this variable at all in my stepdown decision / This variable is considered but not an absolute requirement prior to stepdown / This variable is an absolute requirement prior to stepdown)

**Bacteremic urinary tract infection / pyelonephritis**

- For gram-negative bacteremic urinary tract infection/pyelonephritis in patients that are not severely immunocompromised, what is your typical treatment duration? (Dropbox with numbers 0, 1, 2, 3, 4, …, 28+ days)
  - Please indicate whether the individual criteria below influence treatment duration
    - Antibiotic resistance pattern of pathogen (I do not consider this variable at all in my duration decision / This variable is somewhat considered in my duration decision / This variable is strongly considered in my duration decision)
    - Species of gram-negative bacteria (e.g., *Escherichia coli* versus *Pseudomonas aeruginosa*) (I do not consider this variable at all in my duration decision / This variable is somewhat considered in my duration decision / This variable is strongly considered in my duration decision)
- Do you typically treat with IV antibiotics for the entire course? (yes/no)
  - If no: Is there a minimum duration of IV antibiotics you provide (yes/no)
    - If yes: What is the duration? (number)
  - If no: Please indicate whether individual criteria below influence decision to stepdown to oral therapy
    - Afebrile (I do not consider this variable at all in my stepdown decision / This variable is considered but not an absolute requirement prior to stepdown / This variable is an absolute requirement prior to stepdown)
    - Normotensive (I do not consider this variable at all in my stepdown decision / This variable is considered but not an absolute requirement prior to stepdown / This variable is an absolute requirement prior to stepdown)
    - Concern for poor oral absorption (I do not consider this variable at all in my stepdown decision / This variable is considered but not an absolute requirement prior to stepdown / This variable is an absolute requirement prior to stepdown)
    - Normal WBC (I do not consider this variable at all in my stepdown decision / This variable is considered but not an absolute requirement prior to stepdown / This variable is an absolute requirement prior to stepdown)
    - Available effective agent with high oral bioavailability (I do not consider this variable at all in my stepdown decision / This variable is considered but not an absolute requirement prior to stepdown / This variable is an absolute requirement prior to stepdown)
    - Negative follow-up blood cultures (I do not consider this variable at all in my stepdown decision / This variable is considered but not an absolute requirement prior to stepdown / This variable is an absolute requirement prior to stepdown)
    - Whether gram-negative bacteria is an Enterobacterales (e.g., *Escherichia coli, Klebsiella* species*,* *Enterobacter* species), as opposed to other gram-negative bacteria such as *Pseudomonas aeruginosa* (I do not consider this variable at all in my stepdown decision / This variable is considered but not an absolute requirement prior to stepdown / This variable is an absolute requirement prior to stepdown)

**Bacteremic intra-abdominal infection**

- For gram-negative bacteremic intra-abdominal infections in patients that are not severely immunocompromised, what is your typical treatment duration? (Dropbox with numbers 0, 1, 2, 3, 4, …, 28+ days)
  - Please evaluate whether the individual criteria below influence treatment duration
    - Antibiotic resistance pattern of pathogen (I do not consider this variable at all in my duration decision / This variable is somewhat considered in my duration decision / This variable is strongly considered in my duration decision)
    - Species of gram-negative bacteria (e.g., *Escherichia coli* versus *Pseudomonas aeruginosa*) (I do not consider this variable at all in my duration decision / This variable is somewhat considered in my duration decision / This variable is strongly considered in my duration decision)
    - There is evidence that surgical source control achieved (I do not consider this variable at all in my duration decision / This variable is somewhat considered in my duration decision / This variable is strongly considered in my duration decision)
- Do you typically treat with IV antibiotics for the entire course? (yes/no)
  - If no: Is there a minimum duration of IV antibiotics you provide (yes/no)
    - If yes: What is the duration? (number)
  - If no: Please evaluate whether individual criteria below influence decision to stepdown to oral therapy
    - Afebrile (I do not consider this variable at all in my stepdown decision / This variable is considered but not an absolute requirement prior to stepdown / This variable is an absolute requirement prior to stepdown)
    - Normotensive (I do not consider this variable at all in my stepdown decision / This variable is considered but not an absolute requirement prior to stepdown / This variable is an absolute requirement prior to stepdown)
    - Concern for poor oral absorption (I do not consider this variable at all in my stepdown decision / This variable is considered but not an absolute requirement prior to stepdown / This variable is an absolute requirement prior to stepdown)
    - Normal WBC (I do not consider this variable at all in my stepdown decision / This variable is considered but not an absolute requirement prior to stepdown / This variable is an absolute requirement prior to stepdown)
    - Available effective agent with high oral bioavailability (I do not consider this variable at all in my stepdown decision / This variable is considered but not an absolute requirement prior to stepdown / This variable is an absolute requirement prior to stepdown)
    - Negative follow-up blood cultures (I do not consider this variable at all in my stepdown decision / This variable is considered but not an absolute requirement prior to stepdown / This variable is an absolute requirement prior to stepdown)
    - Whether gram-negative bacteria is an Enterobacterales (e.g., *Escherichia coli, Klebsiella* species*,* *Enterobacter* species), as opposed to other gram-negative bacteria such as *Pseudomonas aeruginosa* (I do not consider this variable at all in my stepdown decision / This variable is considered but not an absolute requirement prior to stepdown / This variable is an absolute requirement prior to stepdown)
    - There is evidence that surgical source control was achieved (I do not consider this variable at all in my stepdown decision / This variable is considered but not an absolute requirement prior to stepdown / This variable is an absolute requirement prior to stepdown)

**Bacteremic skin/soft tissue infection**

- For gram-negative bacteremic skin/soft tissue infections in patients that are not severely immunocompromised, what is your typical treatment duration? (Dropbox with numbers 0, 1, 2, 3, 4, …, 28+ days)
  - Please evaluate whether the individual criteria below influence treatment duration
    - Antibiotic resistance pattern of pathogen (I do not consider this variable at all in my duration decision / This variable is somewhat considered in my duration decision / This variable is strongly considered in my duration decision)
    - Species of gram-negative bacteria (e.g., *Escherichia coli* versus *Pseudomonas aeruginosa*) (I do not consider this variable at all in my duration decision / This variable is somewhat considered in my duration decision / This variable is strongly considered in my duration decision)
- Do you typically treat with IV antibiotics for the entire course? (yes/no)
  - If no: Is there a minimum duration of IV antibiotics you provide (yes/no)
    - If yes: What is the duration? (number)
  - If no: Please evaluate whether individual criteria below influence decision to stepdown to oral therapy
    - Afebrile (I do not consider this variable at all in my stepdown decision / This variable is considered but not an absolute requirement prior to stepdown / This variable is an absolute requirement prior to stepdown)
    - Normotensive (I do not consider this variable at all in my stepdown decision / This variable is considered but not an absolute requirement prior to stepdown / This variable is an absolute requirement prior to stepdown)
    - Concern for poor oral absorption (I do not consider this variable at all in my stepdown decision / This variable is considered but not an absolute requirement prior to stepdown / This variable is an absolute requirement prior to stepdown)
    - Normal WBC (I do not consider this variable at all in my stepdown decision / This variable is considered but not an absolute requirement prior to stepdown / This variable is an absolute requirement prior to stepdown)
    - Available effective agent with high oral bioavailability (I do not consider this variable at all in my stepdown decision / This variable is considered but not an absolute requirement prior to stepdown / This variable is an absolute requirement prior to stepdown)
    - Negative follow-up blood cultures (I do not consider this variable at all in my stepdown decision / This variable is considered but not an absolute requirement prior to stepdown / This variable is an absolute requirement prior to stepdown)
    - Whether gram-negative bacteria is an Enterobacterales (e.g., *Escherichia coli, Klebsiella* species*,* *Enterobacter* species), as opposed to other gram-negative bacteria such as *Pseudomonas aeruginosa* (I do not consider this variable at all in my stepdown decision / This variable is considered but not an absolute requirement prior to stepdown / This variable is an absolute requirement prior to stepdown)
- Would you be willing to enroll your patients with gram-negative bacteremia in a trial of full IV treatment versus PO step down before 72 hours after index blood culture? (Yes/No)
- What are the barriers to enrolling your patients in a trial of IV versus oral stepdown (text box)
